# A Potential Role for *Drosophila* Mucins in Development and Physiology

**DOI:** 10.1371/journal.pone.0003041

**Published:** 2008-08-22

**Authors:** Zulfeqhar A. Syed, Torleif Härd, Anne Uv, Iris F. van Dijk-Härd

**Affiliations:** 1 Department of Medical Chemistry and Cell Biology, Institute of Biomedicine, Göteborg University, Göteborg, Sweden; 2 Department of Medical Genetics, Institute of Biomedicine, Göteborg University, Göteborg, Sweden; Max-Planck-Institut fuer Neurobiologie, Germany

## Abstract

Vital vertebrate organs are protected from the external environment by a barrier that to a large extent consists of mucins. These proteins are characterized by poorly conserved repeated sequences that are rich in prolines and potentially glycosylated threonines and serines (PTS). We have now used the characteristics of the PTS repeat domain to identify *Drosophila* mucins in a simple bioinformatics approach. Searching the predicted protein database for proteins with at least 4 repeats and a high ST content, more than 30 mucin-like proteins were identified, ranging from 300–23000 amino acids in length. We find that *Drosophila* mucins are present at all stages of the fly life cycle, and that their transcripts localize to selective organs analogous to sites of vertebrate mucin expression. The results could allow for addressing basic questions about human mucin-related diseases in this model system. Additionally, many of the mucins are expressed in selective tissues during embryogenesis, thus revealing new potential functions for mucins as apical matrix components during organ morphogenesis.

## Introduction

Epithelia that are in contact with the external environment often produce special molecular structures for apical surface protection. These matrices contain macromolecular assemblies rich in carbohydrate moieties and protect the epithelium from mechanical damage, are barriers against microorganisms and toxic molecules, and help to keep the epithelial surface hydrated and lubricated. Our knowledge about the composition and functions of such apical linings, however, is limited.

A group of large glycosylated macromolecules, important to the mucosal lining of mammalian organs, is the mucin family. Mucins are abundant in vertebrate lungs and digestive tract, and provide lubrication of the luminal surface and protection of the underlying epithelium against physical damage and pathogens [1]. Mucins are either secreted and gel-forming, or attached to the membrane by special cleavable transmembrane domains. The main characteristic of mucin proteins is their extended regions of tandemly repeated sequences that contain prolines together with serines and/or threonines, to which large sugar side chains attach [2]. These PTS (proline, threonine and serine)-repeats generally occupy between 30 and 90% of protein length and are envisioned as an outstretched polypeptide backbone densely covered with carbohydrate moieties much like a bottlebrush. The remaining parts of the protein often contain conserved protein domains that mediate protein-protein interactions. Thus, mucins are capable of forming enormous networks, to which the glycosylated PTS repeats confer high water-binding capacity, a selective barrier function and the ability to trap microorganisms.

Mucin-like proteins have so far been poorly characterized in non-mammalian organisms. The PTS repeats cannot be detected in homology searches due to their poor sequence conservation, and biochemical mucin isolation is hampered by the heavy glycosylations, which makes the proteins large and difficult to extract. Moreover, the repetitive PTS repeats are sparsely represented in cDNA libraries. Recently, a bioinformatics search for proteins that contain mucin-associated domains combined with a subsequent survey for PTS repeats (based on their high ST-content) identified putative gel-forming mucins in several divergent species, including frog, zebra-fish and the starlet sea anemone [3]. It is therefore plausible that mucins have a broader function across species and organs than previously anticipated.

Here, we identify and characterize PTS repeat containing proteins in *Drosophila melanogaster*. In an initial BLAST search for *Drosophila* proteins that contain mucin-associated domains, we found that very few of those also contain PTS repeats. To identify *Drosophila* mucins, we thus devised a strategy to directly recognize the PTS repeats based on a combination of content and pattern homology. Using this approach we find that the predicted *Drosophila* proteome contains more than 30 proteins with extended PTS repeats. The temporal and spatial expression patterns of transcripts corresponding to 23 of these mucin-type proteins suggest that they not only have analogous functions to vertebrate mucins, but also are novel components of yet uncharacterized molecular assemblies that may be important for organ development.

## Results

### Identification of *Drosophila* mucins

Forty-two *Drosophila* proteins have a serine and threonine content of more than 25% and at least four repeats of ten amino acids (Table S1). When subjected to manual sequence analysis, a large number (thirty-three) of these proteins turned out to contain mucin-like PTS repeats (Table S2). The remaining nine proteins contain stretches of only serine or threonine, or repeats with other amino acids than serine and threonine. The 33 proteins were further classified depending on the content and relative size of the repeat domains. Sixteen proteins contained PTS repeats that constitute more than one-third of protein length, and were named mucins (Muc). The remaining 17 proteins contained repeats shorter than one-third of protein length, or without prolines, and were termed mucin-related-proteins (Mur). In addition, three proteins with low serine and threonine content, but with mucin-like PTS repeats, were identified during initial homology searches for mucin-associated domains (CG33196 and CG13648) and chitin-binding domains (CG32656) and classified as above (Table S2B). Thus, in total seventeen *Drosophila* mucins and nineteen mucin-related proteins were identified.

### *Drosophila* mucins and mucin-related proteins

**Figure 1:**
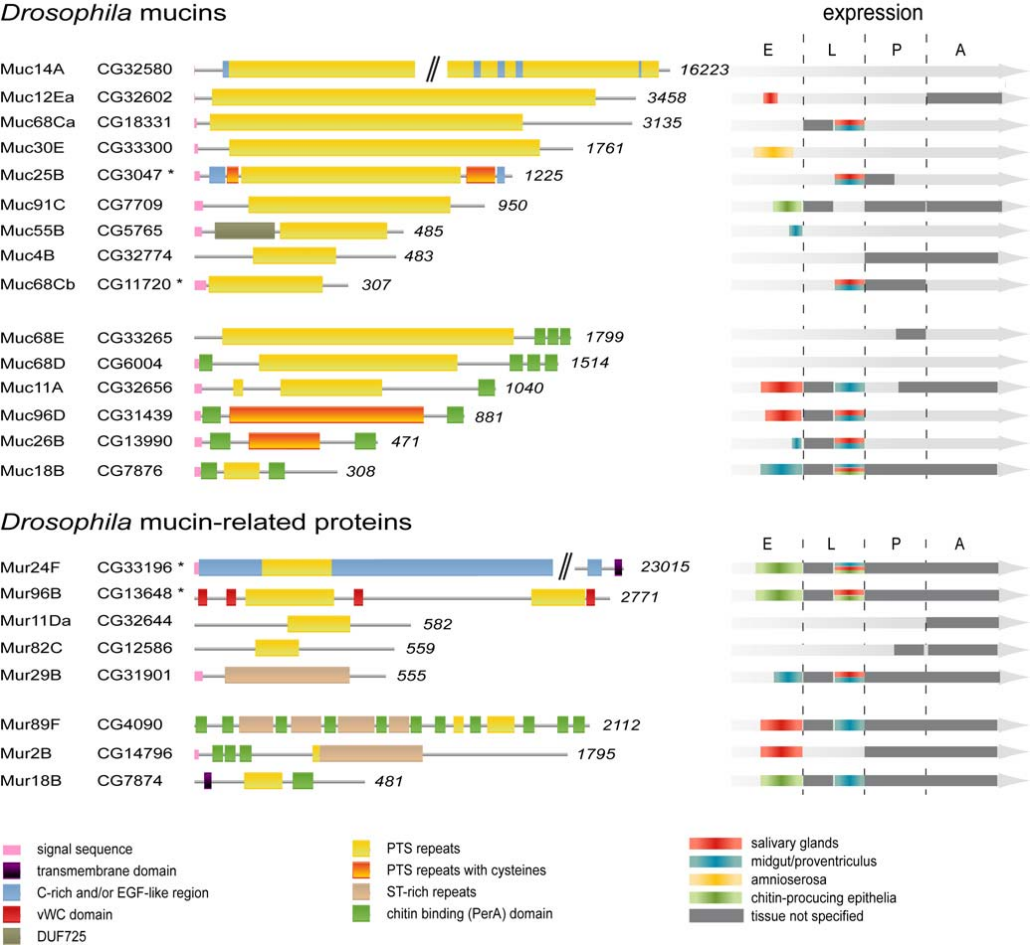
*Drosophila* mucins and mucin-domain containing proteins. The proteins that were analyzed in this study are illustrated (left) together with an overview of their stage and dominant tissue-specific expressions (right). Conserved protein domains were predicted using the EMBL-database and are shown together with the identified PTS repeats according to color code (bottom). In proteins classified as mucins, the PTS repeats (yellow = without cysteines, orange = with cysteines) make up at least one-third of total protein length. Proteins in which the PTS repeats constitute less than one-third of protein length, lack proline (brown) or have low ST content (Mur11-A2) were termed mucin-related proteins. Each protein was named according to protein class (Muc: Mucins and Mur: Mucin-related proteins) followed by the cytological position. Proteins marked with “*” were previously identified as Sgs1 (Muc25B), Sgs3 (Muc68Cb), Dp (Mur24F) and Tnc (Mur96B). The spatial and temporal expression of each Muc and Mur were compiled from embryonic *in situ* hybridizations (E), RT-PCR on larvae (L; first instar larvae to the left and dissected third instar larval organs to the right), RT-PCR on pupae (P) and RT-PCR on adults (A). Where applicable, the dominant organ-specific expression is illustrated by color code. (An extensive overview of expression data is given in Table S4).

Fifteen mucins and eight mucin-related proteins were further characterized in this study (Table S3). Domain analyses of the proteins show that nine of these contain peritrophin A (PerA) chitin-binding domains (Figure 1). Among the remaining mucins and mucin-related proteins, only five proteins contain conserved domains in addition to their repeats. Muc14A, Muc25B (salivary gland protein 1; Sgs1) and Mur24F (Dumpy, Dp [4]) harbour stretches of C-rich EGF-like regions that may mediate protein-protein interactions. Mur96B (Tenectin, Tnc [5]) contains four predicted protein-protein interaction domains of the von Willebrand factor C type and Muc55B contains a *drosophila*-specific domain of unidentified function (DUF725). The length of the largest *Drosophila* mucin identified, Muc14A, approaches that of human MUC16. In three of the proteins the PTS repeats themselves contain cysteines, as previously shown for Xenopus [6]. Interestingly, a significant number of the mucins and mucin-related proteins (6 and 3, respectively) lack conserved domains and thus appear to function solely through their extensive PTS repeat.

### *Drosophila* mucins are expressed at different stages of the fly life cycle

**Figure 2:**
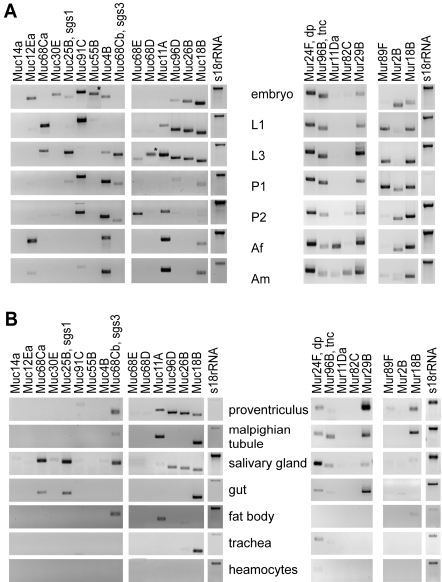
*Drosophila* mucins and mucin-domain containing proteins are expressed throughout development. Transcripts correlating to mucins and mucin-related proteins were detected by two-step RT-PCR (Reverse Transcription–PCR). A) Template RNA was isolated from embryos (E), first instar larvae (L1), third instar larvae (L3), early pupae (P1), late pupae (P2), adult females (Af) and adult males (Am), and the products separated on agarose gels. Primers for the 18S-rRNA gene were used as control. Samples imported from another gel are indicated with “*”. Multiple bands (as observed for *Mur29B*, *Mur2B* and *Mur18B* products) most likely arise from multiple priming sites, due to the repetitive nature of the gene sequences. B) Detection of transcripts in dissected larval organ was assessed by two-step RT-PCR. As positive control, primers for the 18S-rRNA gene were used.

To gain insight into possible functions of the identified mucins and mucin-related proteins, we addressed their expression-levels during different stages of the fly life cycle. Reverse transcription (RT) PCR was performed on RNA extracts from embryos, first instar larvae, third instar larvae, early pupae, late pupae and adults. If a mucin participates primarily in functional, non-developing organs, its expression would be expected to rise in larvae and adults, as compared to the preceding stages of embryogenesis and metamorphosis. Instead, we found that mucins and mucin-related proteins are dynamically expressed both during developmental phases and in the physiologically active organism (Figure 2A). Exceptions to this are the mucin *Muc30E*, which was detected clearly only during embryogenesis, *Muc68D* that was present only in the larvae, and *Mur11Da*, which was only expressed in adults.

### *Drosophila* mucins are expressed in cuticle-free organs

If the physiological functions of *Drosophila* mucins correlate with those of vertebrate mucins, they should localize to the lumen surface of organs that are in contact with the external environment. As many *Drosophila* organs are protected by cuticle, the primary sites of mucin expression are expected to reside in the cuticle-free salivary glands, midgut and renal tubes (malpighian tubules). RT-PCR on selected organs from third instar larvae indeed recognized a significant portion of mucin transcripts in these tissues (Figure 2B). Nine of the analyzed genes were detected in third instar larval salivary glands and eight of those were also expressed in the third instar larval gut and/or proventriculus. Three mucins were expressed exclusively in the digestive tract and seven were expressed in malpighian tubules, most of which also were expressed in both salivary glands and proventriculus. Only four genes were expressed in the larval fat body and just one, *Mur24F/Dp*, showed very faint expression in hemocytes.

### Mucins are widely expressed during *Drosophila* embryonic development

**Figure 3:**
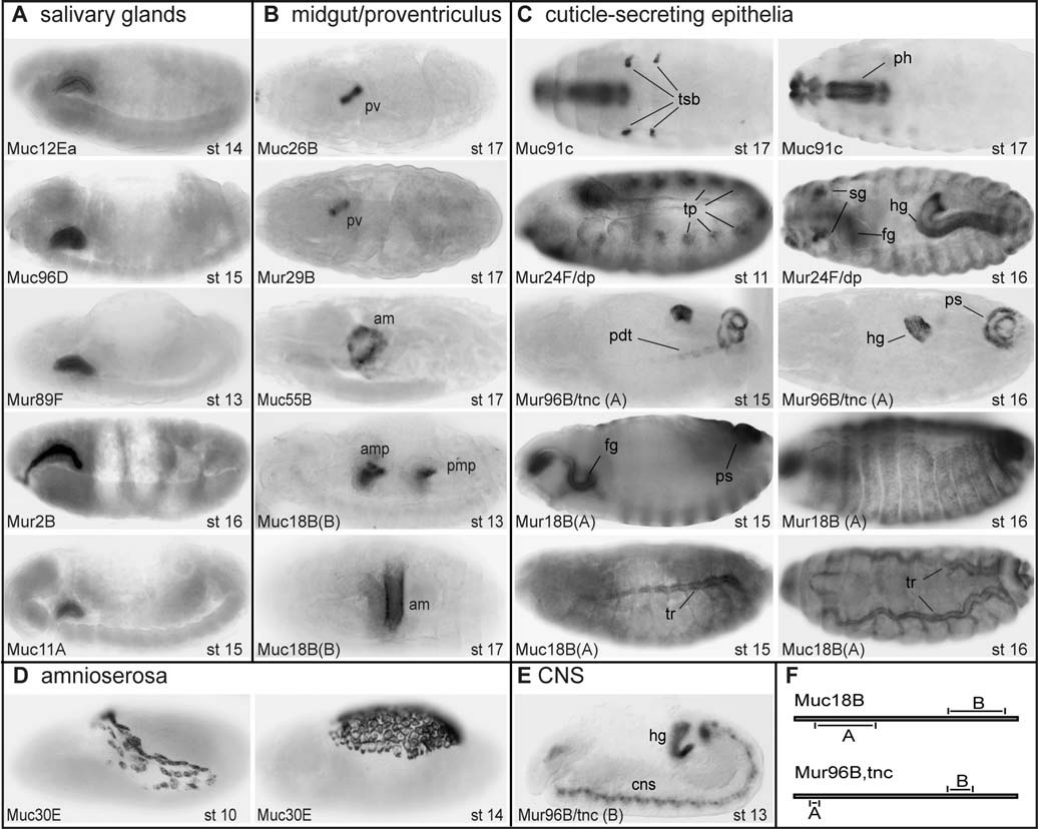
Embryonic expression of *Drosophila* mucins and mucin-related proteins. The embryonic expression pattern of each mucin and mucin-domain containing protein was detected by RNA *in situ* hybridizations on whole-mount embryos. A) Genes expressed in the salivary glands include *Muc12Ea* at stages 14 and 15, as well as *Muc96D Mur89F*, *Mur2B* and *Mur11A* from stage 13. All embryos are lateral views, anterior to the left. B) The expression of four genes was detected in the digestive tract. *Muc26B* and *Mur29B* are expressed in the late proventriculus (pv; stage 16/17; ventral view), whereas *Muc55B* (lateral view) and *Muc18B* (probe B; ventral view) are detected in the anterior midgut (am). *Muc18B* (probe B) is also expressed in the developing gut from stage 13, where it is seen in the anterior and posterior midgut primordia (amp and pmp; lateral view). C) Ectoderm-derived cells that will produce cuticle express mucins and mucin-domain containing proteins either before or after cuticle formation. *Muc91C* is detected only in late pharynx (ph; stage 17) and tracheal spiracular openings (tsb; dorsal views), but *Mur24F*, *Mur96B* and *Mur18B* (probe A) are expressed in ectodermal cells already prior to cuticle production, such as in the tracheal pits (trp), tracheal prosterior dorsal trunk (pdt), foregut (fg), hindgut (hg) and around the posterior spiracles (ps) (*Mur24F* stage 16 is dorsal view, the others lateral views). D) *Muc30E* is expressed exclusively in the amnioserosa throughout development (lateral views). E) One of the genes, *Mur96B/tnc*, is also detected in the central nervous system (CNS) when using probe B (lateral view). F) Probes directed against distinct parts of the *Mur96B* and *Muc18B* coding regions (A and B, as illustrated) gave different expression patterns.

Since 15 of the mucins were expressed during embryogenesis, we performed whole-mount RNA *in situ* hybridizations to address their tissue localization at this developmental stage (Figure 3). We found that five mucins were expressed in the developing salivary gland (Figure 3A) at a time point corresponding to the presence of a luminal matrix that is detected by antiserum against O-linked GalNAc [7]. Similarly, three mucins (*Mur96B/tnc*, *Mur24F/dp* and *Mur18B*) showed predominant expression in the developing fore- and hindgut and the trachea, when these organs are temporarily filled with O-glycan-rich material (Figure 3C). *Muc55B* and *Muc18B* (probe B) were detected early in the developing embryonic midgut (Figure 3B), while transcripts for *Muc26B* and *Mur29B* were found in the proventriculus at later stages (from stage 16).

Mucin expression was also evident in cuticle-producing epithelia. The cuticle is a multilayered matrix that is in close contact with the apical epithelial surface. *Mur96B/tnc*, *Mur24F/dp* and *Mur18B* were detected in the epidermis before, during and after cuticle production (figure 3C). In contrast, *Muc91C* and *Muc18B* both were expressed only at the time of cuticle secretion (from late stage 15, Figure 3C). The large transmembrane *Mur24F/dp* was expressed in all cuticle-producing tissues, while the mucins were expressed only in a subset of cuticle producing cells.

Some mucin expression patterns fell outside that of tubular organ and cuticle producing epithelia. *Muc30E* was expressed selectively in the extra-embryonal amnioserosa (figure 3D) that covers the dorsal side of the embryo before the epidermis closes at the dorsal midline, and a *Mur96B/tnc* isoform (Figure 3E) was detected in the central nervous system (CNS).

## Discussion

In the current study, we have identified a large mucin-like family of *Drosophila* proteins based on the characteristics of their extensive PTS repeats. Lang et al [8] previously targeted PTS repeats to identify mucins. They used “Mpred”, a Hidden Markov model that decides if an amino acid sequence conforms to a mucin domain, and “PTSpred”, an algorithm based on amino acid compositional bias. In our study we search the predicted protein database for proteins with a total ST-content above threshold level, combined with a requirement for at least 4 repeats within the protein. Thus, using the thresholds recently used by Lang et al [3] (an ST-content of at least 20% and a P content of 5%) our program recognized 67 *Drosophila* repeat-containing proteins, only 12 of which were identified by their approach. Five proteins from that study were not identified in our search due to lack of repetitive sequences (CG8181, CG3280, CG15765, CG17211), or an ST-content lower than 20% (CG14120). The combination of two parameters in one program provides extra stringency to the predictions and reduces false positives (see Table S1 for results without the repeat requirement). Yet, the high number of proteins identified shows that sensitivity is retained. Additionally, the use of a first step that restricts the size of the database, allows faster performance of the following step that requires more computer capacity. Once the search criteria were established, the program delivered the output for the *Drosophila* proteome overnight, and we are currently extending our searches to include other *Drosophila* species (results of which will be available at http://www.biomedicine.gu.se/drosophila when completed).

Biochemical approaches for identification of glycosylated proteins in *Drosophila* have also been described. Many proteins with mucin-type O-glycosylation were identified by a serial lectin affinity-purification of S2 cell proteins [9], but none of these complied to the criteria used in our search. In our analysis *Drosophila* appears to lack transmembrane mucins and S2 cells are therefore not expected to produce mucin-like proteins. A different proteomic analysis of the hemolymph clot resulted in the description of 4 mucin-type proteins, 2 of which are recognized by our PTSP-Miner (CG7604/Eig71E/Muc71E [10], [11] and CG15825 [12]). Finally, a third approach aimed to identify peritrophins and used the expected chitin-binding properties of such proteins for affinity-purification. All of the peritrophins recognized contain a PerA domain, but only one harbors PTS repeats [13] and was identified also by our method (CG13990/Muc26B).

Protein-protein-interaction domains are a prerequisite for vertebrate mucin gel formation. The PerA domains found in many *Drosophila* mucins and mucin-related proteins may represent an adaptation to the insect-specific chitin-containing molecular structures and confer analogous complex or gel-forming properties by binding to chitin chains [14]. Moreover, three of the identified mucins contain cysteines within their PTS repeats, which previously were suggested to organize mucin chain interactions in Xenopus similar to the cysteine-rich CysD domains of human gel forming mucins [3]. Thus, although *Drosophila* mucins lack the characteristic mucin-associated domains of known vertebrate mucins, they do have potential to form analogous gel forming matrices via other protein properties, and it will now be possible to address that question for each of the identified proteins.

Consistent with a protective function for *Drosophila* mucins on organ epithelial surfaces, we found that the majority of the identified mucins are expressed by cuticle-free epithelia in larvae. In insects, a Peritrophic Matrix (PM) that contains chitin fibers and glycosylated proteins protects the digestive tract [14], [15]. The current study identifies twelve digestive tract mucins (Table S4). Their embryonic expression patterns suggest that some of these are integral components of the PM and thus may represent novel insect peritrophins. Indeed, a previously reported PM protein, the Invertebrate Intestinal Mucin (IIM) from *T. ni* larvae [16], is homologous to Muc26B identified in this study. Furthermore, four of the digestive tract mucins were expressed in the late embryo at either the anterior midgut or at a specific region within the proventriculus, which are sites that correlate with type I and type II PM production, respectively (Figure 3B). By targeting the expression of these mucins it should now be possible to address their contribution to insect immunity in natural *Drosophila* infection models and their function in maintenance of the selective PM barriers.

In addition to the digestive tract, a second *Drosophila* tissue with prominent mucin expression is the salivary gland. Further studies will have to show if these mucins are glue components, like the previously identified Muc25B/sgs1 and Muc68Cb/sgs3, or if they serve to lubricate and protect the apical salivary gland epithelium, like the human salivary gland secreted mucins (MUC5B, MUC7). Some of the salivary gland mucins harbor perA domains, despite the absence of chitin production by salivary glands. The presence of the chitin-binding domains might simply be explained by the fact that all of those salivary gland mucins also are expressed in the chitin-containing digestive tract. Alternatively, the perA domains may interact with GlcNAc residues on glycoproteins and glycolipids, or with PM-chitin upon ingestion together with food-intake. The finding that *Drosophila* mucins are expressed in organs similar to those of their vertebrate counterparts, namely the digestive tract and salivary glands, could open up for addressing basic questions about human mucin-related diseases in this genetically advanced model system.

An interesting finding was the abundant expression of mucins in the developing embryo. As their expression patterns correlate with that of apical and luminal matrices detected by antisera and lectins that detect the typical mucin-type O-glycosylations [7], these proteins may represent new components of such matrices. The only characterized apical matrices in *Drosophila* embryos are chitin-containing and include a temporary luminal matrix that is required to shape tracheal tubes [17]. It is an exciting possibility that large glycosylated proteins, like mucins, similarly contribute to the shaping of non-chitin-producing organs by providing a luminal scaffold during their development. Indeed, anti-Tnc/Mur96 labeling has previously revealed that the Tnc/Mur96 protein is present within the tracheal lumen from stage 15 and along the apical surface of the fore- and hindgut [5]. Of interest for further studies in this context may also be the mucins expressed in the developing salivary gland, since a defective secretory content of the lumen has been associated with regions of abnormal tube dilations and constrictions [18].

Expression data for the UDP-GalNAc:polypeptide N-acetylgalactosaminyltransferases (pgants) that initiate mucin-type O-glycosylation [19] parallel the observed mucin expression patterns in *Drosophila* embryos. This further supports that the PTS repeats of the identified proteins could act as substrates for O-glycosylation. Organ-restricted mucin expressions correlate with expression of specific pgants, for example Muc30E and CG30463 in the amnioserosa, Muc91C and pgant3 in pharynx and Muc26B/Mur29B and pgant4 in the proventriculus. Additionally, expression of core-1 ß1-3 galactosyltransferases is present in the amnioserosa (CG9520) and late salivary glands (CG9520, CG8708, CG13904-1; [20]). A future challenge is to confirm and determine the actual glycosylation for the identified mucins during different developmental stages.

The current characterization of *Drosophila* mucins should make it possible to address different functional aspects for each of the identified proteins. The results also provide a means to investigate the importance of apical matrices, mucins and mucin-type glycosylation for various physiological and developmental processes, using the genetic tools and advantages available for *Drosophila*.

## Materials and Methods

### Identification of *Drosophila* mucins

BLAST (EMBL, Flybase) and domain searches (SMART) were performed to identify *Drosophila* proteins that contain the mucin-associated SEA, vWD4, EGF and cystein-knot domains, and identified proteins were manually scanned for the presence of PTS repeats. Next, we developed a bioinformatic program using Java programming language and BioJava subroutines [21] to directly mine the whole predicted protein database for the presence of PTS repeats. The first step in the program is implemented to calculate the total frequency of the amino acids, serine (S), threonine (T) and proline (P) in a given protein sequence, and the second step identifies the number of amino acid repeats in this sequence. We thus call the program the PTSP-Miner (PTS Pattern-Miner; available at http://www.biomedicine.gu.se/drosophila). A more detailed description is given in supplementary material (Text S1).

### Nomenclature of identified proteins

The PTSP-Miner output proteins were classified according to the nature of their tandem repeats. Thus, sixteen proteins, in which the PTS repeats occupy at least one third of total protein length, were defined Mucins. Of the remaining 25 proteins, 17 proteins either have a shorter PTS domain or only have ST-rich repeats (i.e, lack prolines) and those were defined as mucin-related, whereas 9 proteins did not contain a repeat region (“false positives” in Table S2). Four of the identified proteins have previously been named Tenectin, Dumpy, Sgs1 and Sgs3. In this study, we adopted a simple nomenclature for all proteins, where mucins were named Muc and the mucin-related proteins were named Mur, followed by the cytological position at which they are encoded. If multiple genes are present in the same position, the name is followed by a, b, etc.

### *In Situ* Hybridization

To establish the expression pattern of the identified mucins and mucin-domain containing proteins RNA *in situ* hybridization on whole-mount embryos was performed as described in Tonning et al 2006 [17]. In short, embryos were collected for 18 hours (age = 0 to 18 hours after egg laying), dechorionated and fixed in 4% formaldehyde. After devitellinization, the embryos were re-fixed, washed and rinsed in PBT:Hybridization buffer (Foramide, 20× SSC, Tween-20, ssDNA (2 mg/ml) Heparin (10 mg/ml)). Prehybridization was performed in hybridization buffer at 70°C for 2 hours. RNA probes were synthesized using the DIG RNA labeling kit (Roche Applied Science), according to the instructions of the manufacturer. Primers used for probe synthesis are listed in Table S5. Hybridization was performed with DIG-labeled sense and anti-sense RNA probes at 56°C overnight in water bath. After post-hybridization washes the embryos were incubated with Anti-Digoxigenin-AP Fab fragments (Roche) 1∶2000 in PBSBT (PBS plus 0.1% Triton X-100 and 0.2% BSA) overnight at 4°C and transcripts were visualized through a color reaction using NBT and BCIP (Roche). Embryos were suspended in 70% glycerol and mounted.

### Reverse transcription and PCR

The RT-PCR was performed according to a two-step protocol. In short, embryos, larvae, pupae, adult flies and third instar larval organs were homogenized with an Eppendorf homogenizer (Kontes glass company, New Jersey) and RNA was prepared and DNAse-treated on a mini-column using Qiagen's Rneasy kit. Reverse transcription to generate cDNA was performed using Invitrogen's RT-kit. The PCR was run with Platinum Taq Polymerase (Invitrogen) in a 15 µl reaction with the following program: 95°C 1 min, then 35 cycles 94°C 30 sec, 58°C 30 sec, 72°C 30 sec, followed by a 5 min extension at 72°C. When possible, the PCR primers were designed so that the PCR-product spans an intron, to detect any product arising from contaminating DNA. The sequences of all primer pairs are listed in supplementary data (Table S5). Negative controls, in which the reverse transcriptase was excluded, were included for each PCR, and those samples that still contained DNA (probably due to the existence of polytene genes in *Drosophila*) were treated a second time with DNase.

## Supporting Information

Text S1 A detailed description of the PTSP-Miner.
(0.04 MB DOC)

Table S1 PTSP-Miner output using different cutoff values. The number of Drosophila proteins identified differs when using two different threshold levels for total serine and threonine content (20% and 25%), and when adding a repeat criterium, but not with three cutoff values for proline content (5%, 1% and 0.1%). The cutoff values used in the analysis are outlined, whereas all other raw output data can be found at (http://www.biomedicine.gu.se/drosophila).
(0.04 MB DOC)

Table S2 Identified Drosophila mucins and mucin-domain containing proteins. The results of the PTSP-Miner applied to the Drosophila annotated protein database (version 42.43). A) The predicted proteins selected by the PTSP-Miner when the ST-content cutoff = 25%, total peptide length >300 amino acids, P-content >0.1% and number of ten amino acids-repeats >3. The reason for defining proteins as mucin-domain containing proteins and not as mucins is given in the rightmost column. Proteins listed under the heading “false positives” do not contain PTS repeat domains, but instead, they contain either stretches of serine or threonine only, or repeats without those residues. B) Drosophila proteins identified by scanning proteins that contain other mucin-associated domains for PTS domains. The domain by which the protein was identified is listed in the rightmost column. C) The PTSP-Miner raw output data for Drosophila using other cutoff values are available at (http://www.biomedicine.gu.se/drosophila).
(0.13 MB DOC)

Table S3 Analysis of Drosophila mucins and mucin-related proteins. The amino acid residues predicted to function as signal sequences (SS) and the start of transmembrane domains (TM) are indicated for each protein. The serine/threonine content (ST%), proline content (P%), and the size of the repeat domain (RD) of each protein are presented as percentage of entire protein length and as absolute length in amino acids. Sgs = salivary glue protein, Dp = dumpy, Tnc = tenectin,
(0.07 MB DOC)

Table S4 Overview of Drosophila mucins and mucin domain-containing proteins expression profiles. Expression at a certain stage is indicated by “+”, where the color specifies the organ of expression according to the color code at the bottom of the table. Weak expression is indicated by “(+)”. The data were collected from embryonic in situ hybridizations at stages 12–17 (ISH) and RT-PCR (from all stages as well as from L3 organs). Template RNA for the RT-PCR was isolated from embryos (E), first instar larvae (L1), third instar larvae (L3), early pupae (P1), late pupae (P2), adult females (Af) and adult males (Am).
(0.04 MB XLS)

Table S5 List of oligos used for RT-PCR and for synthesis of the RNA in situ probes. For each gene, we used the same set of oligos for Reverse Transcription PCR and for amplification of the template DNA used to generate the in situ probes. When two different oligo pairs (A and B) are presented for a single gene, it means that the products yielded differential expression patterns in RNA in situ hybridization.
(0.08 MB DOC)
