# Supplementary material for: A Potential Role for Drosophila Mucins in Development and Physiology
Source: PLoS One. 2008 Aug 22;3(8):e3041. doi: 10.1371/journal.pone.0003041 (PMC2515642; doi:10.1371/journal.pone.0003041)
Supplement: Text S1 — A detailed description of the PTSP-Miner. (0.04 MB DOC) [file pone.0003041.s001.doc]

The PTSP-Miner runs on all platforms supporting Java and requires Java runtime environment (JRE) 1.5 and BioJava 1.5 libraries. The *Drosophila* *melanogaster* protein sequence database was downloaded in FASTA format from Ensembl, available online at (www.ensembl.org), database version 42.43. In detail, the data in this study were obtained by letting the PTSP-Miner score protein sequences with S+T-content of more than 25%, P-content of more than 0.1% and a total protein length greater than 300 amino acids. (Output data with other cutoff values are summarized in Table S1.) The output from this first step (98 proteins) is processed into FASTA format and redirected as an input for the second step, in which the program searches for the occurrence of at least four repeats of ten amino acids. This is achieved by virtually breaking each protein into ten amino acid peptides, each of which is used as a motif in a pattern search against the total protein. The pattern program also provides the total number of repeats for each 10 amino acid stretch. In the final output (42 proteins, all encoded by different genes), each repeat is color-coded so that the real length of repeat, which may combine several consecutive ten amino acid stretches, is visualized. The PTSP-Miner and its raw output data for *Drosophila melanogaster* are available at (http://www.biomedicine.gu.se/drosophila).
