# Supplementary material for: A Potential Role for Drosophila Mucins in Development and Physiology
Source: PLoS One. 2008 Aug 22;3(8):e3041. doi: 10.1371/journal.pone.0003041 (PMC2515642; doi:10.1371/journal.pone.0003041)
Supplement: Table S1 — PTSP-Miner output using different cutoff values. The number of Drosophila proteins identified differs when using two different threshold levels for total serine and threonine content (20% and 25%), and when adding a repeat criterium, but not with three cutoff values for proline content (5%, 1% and 0.1%). The cutoff values used in the analysis are outlined, whereas all other raw output data can be found at (http://www.biomedicine.gu.se/drosophila). (0.04 MB DOC) [file pone.0003041.s002.doc]

| PTS Criteria | Repeat search | Genes | Proteins |
| --- | --- | --- | --- |
| ST>25% P>5% | - | 37 | 46 |
| ST>25% P>5% | + | 30 | 30 |
| ST>25% P>1% | - | 81 | 96 |
| ST>25% P>1% | + | 41 | 41 |
| ST>25% P>0.1% | - | 84 | 98 |
| ST>25% P>0.1% | + | 42 | 42 |
| ST>20% P>5% | - | 339 | 464 |
| ST>20% P>5% | + | 67 | 74 |
| ST>20% P>1% | - | 427 | 600 |
| ST>20% P>1% | + | 86 | 97 |
| ST>20% P>0.1% | - | 424 | 602 |
| ST>20% P>0.1% | + | 87 | 98 |
