# Supplementary material for: A Potential Role for Drosophila Mucins in Development and Physiology
Source: PLoS One. 2008 Aug 22;3(8):e3041. doi: 10.1371/journal.pone.0003041 (PMC2515642; doi:10.1371/journal.pone.0003041)
Supplement: Table S2 — Identified Drosophila mucins and mucin-domain containing proteins. The results of the PTSP-Miner applied to the Drosophila annotated protein database (version 42.43). A) The predicted proteins selected by the PTSP-Miner when the ST-content cutoff = 25%, total peptide length >300 amino acids, P-content >0.1% and number of ten amino acids-repeats >3. The reason for defining proteins as mucin-domain containing proteins and not as mucins is given in the rightmost column. Proteins listed under the heading “false positives” do not contain PTS repeat domains, but instead, they contain either stretches of serine or threonine only, or repeats without those residues. B) Drosophila proteins identified by scanning proteins that contain other mucin-associated domains for PTS domains. The domain by which the protein was identified is listed in the rightmost column. C) The PTSP-Miner raw output data for Drosophila using other cutoff values are available at (http://www.biomedicine.gu.se/drosophila). (0.13 MB DOC) [file pone.0003041.s003.doc]

| **A) Output PTSP-miner** | | | | |  |  | |  |
| --- | --- | --- | --- | --- | --- | --- | --- | --- |
| **Cutoff values: ST25%, P 0,1%, aa >300, rpt >3** | | | | |  |  | |  |
|  |  |  |  |  |  |  | |  |
| **Mucins** |  |  |  |  |  |  |  | |
| **Gene ID** | **total ST-content (%)** | **total P-content (%)** | **total protein length (aa)** | **no. of repeats** | **protein name according to this study** |  |  | |
| CG31439-PA | 70.7 | 5.0 | 881 | 291 | Muc96D |  |  | |
| CG3047-PA | 59.8 | 14.1 | 1286 | 390 | Muc25B, Sgs1 |  |  | |
| CG32774-PA | 50.7 | 9.5 | 483 | 10 | Muc4B |  |  | |
| CG18331-PA | 49.1 | 2.8 | 3135 | 262 | Muc68Ca |  |  | |
| CG11720-PA | 44.0 | 14.7 | 307 | 28 | Muc68Cb, Sgs3 | |  | |
| CG33300-PA | 43.7 | 9.3 | 1701 | 209 | Muc30E |  |  | |
| CG6004-PB | 42.5 | 6.0 | 1514 | 440 | Muc68D |  |  | |
| CG32602-PA | 38.1 | 15.2 | 3269 | 2875 | Muc12Ea |  |  | |
| CG13990-PA | 37.4 | 9.8 | 471 | 90 | Muc26B |  |  | |
| CG32580-PA | 35.3 | 4.2 | 16223 | 10364 | Muc14A |  |  | |
| CG33265-PA | 32.9 | 7.6 | 1799 | 626 | Muc68E |  |  | |
| CG7709-PA | 32.3 | 19.5 | 950 | 74 | Muc91C |  |  | |
| CG32603-PA | 28.7 | 9.3 | 345 | 45 | Muc12Eb |  |  | |
| CG5765-PA | 28.7 | 14.6 | 485 | 87 | Muc55B |  |  | |
| CG7604-PA | 27.2 | 16.6 | 445 | 33 | Muc71E |  |  | |
| CG7876-PA | 26.9 | 8.1 | 308 | 25 | Muc18B |  |  | |
|  |  |  |  |  |  |  |  | |
| **Mucin-domain containing proteins** | | | |  |  |  |  | |
| **Gene ID PTS-containing** | **total ST-content (%)** | **total P-content (%)** | **total protein length (aa)** | **no. of repeats** | **protein name according to this study** | **Excluded as mucin because:** |  | |
| CG31901-PA | 54.4 | 1.3 | 555 | 136 | Mur29B | no P in repeats |  | |
| CG32644-PB | 43.7 | 8.8 | 582 | 23 | Mur11Da | PTS<1/3 |  | |
| CG4090-PA | 35.7 | 7.4 | 2112 | 235 | Mur89F | no P in repeats |  | |
| CG10911-PA | 34.8 | 2.2 | 359 | 25 | Mur55B | no P in repeats |  | |
| CG14796-PA | 33.3 | 10.1 | 1795 | 6 | Mur2B | PTS<1/3 |  | |
| CG32642-PC | 32.8 | 9.2 | 381 | 46 | Mur11Db | no P in repeats |  | |
| CG4835 | 32.3 | 10.5 | 1175 | 11 | Mur64D | PTS<1/3 |  | |
| CG17104 | 31.6 | 6.9 | 449 | 6 | Mur32Aa | PTS<1/3 |  | |
| CG13560-PA | 31.6 | 3.8 | 317 | 13 | Mur59F | no P in repeats |  | |
| CG12586-PA | 30.9 | 13.2 | 559 | 21 | Mur82C | PTS<1/3 |  | |
| CG7290-PA | 29.4 | 3.3 | 419 | 9 | Mur77A | no P in repeats |  | |
| CG40323-PB | 27.1 | 7.9 | 5122 | 2955 | - | *1 |  | |
| CG1433 | 26.6 | 4.1 | 725 | 7 | Mur83B, Atu | PTS<1/3 |  | |
| CG15226-PA | 26.1 | 6.7 | 418 | 8 | Mur57B | PTS<1/3 |  | |
| CG7874-PA | 26.0 | 7.5 | 481 | 26 | Mur18B | PTS<1/3 |  | |
| CG32972-PA | 25.9 | 9.2 | 1853 | 54 | Mur35A | PTS<1/3 |  | |
| CG7300-PB | 25.3 | 0.3 | 886 | 41 | Mur32Ab | no P in repeats |  | |
|  |  |  |  |  |  |  |  | |
| **false positives** | |  |  |  |  |  |  | |
| CG8181-PA | 40.1 | 9.5 | 746 | 11 |  | no PTS |  | |
| CG3918-PA | 31.2 | 3.1 | 321 | 5 |  | no PTS |  | |
| CG33526-PC | 28.8 | 4.9 | 593 | 7 |  | S-strings |  | |
| CG32676-PA | 27.5 | 4.7 | 1122 | 7 |  | no PTS |  | |
| CG2989-PA | 26.4 | 6.0 | 4498 | 6 |  | S-trich, no clear rpt |  | |
| CG11073-PA | 26.0 | 12.8 | 1056 | 5 |  | T-rich, no rpts |  | |
| CG13316-PB | 25.2 | 5.6 | 595 | 13 |  | S-strings |  | |
| CG3563 | 25.4 | 5.4 | 1153 | 4 |  | no PTS |  | |
| CG15721 | 26.6 | 9.7 | 843 | 9 |  | no PTS |  | |
|  |  |  |  |  |  |  |  | |
| **B) Drosophila mucin-domain containing proteins identified by non-PTS domains** | | | | | | | | |
| **Gene ID PTS-containing** | **total ST-content (%)** | **total P-content (%)** | **total protein length (aa)** | **no. of repeats** | **protein name in this analyses** | **Identified by** | |  |
| CG33196 | 16.3 | 12.5 | 23015 | 33 | Mur24F, Dp | EGF domain | |  |
| CG13648 | 20.4 | 11.2 | 2771 | 4 | Mur96B, tnc | VWC domain | |  |
| CG32656 | 17.9 | 15.2 | 1040 | 14 | Muc11A | PerA domain | |  |

1*: CG40323 encodes only repeats, which makes it difficult to perform RT-PCR analysis, and lacks a predicted signal sequence, suggesting that the gene might be a pseudogene.
