# Supplementary material for: A Potential Role for Drosophila Mucins in Development and Physiology
Source: PLoS One. 2008 Aug 22;3(8):e3041. doi: 10.1371/journal.pone.0003041 (PMC2515642; doi:10.1371/journal.pone.0003041)
Supplement: Table S3 — Analysis of Drosophila mucins and mucin-related proteins. The amino acid residues predicted to function as signal sequences (SS) and the start of transmembrane domains (TM) are indicated for each protein. The serine/threonine content (ST%), proline content (P%), and the size of the repeat domain (RD) of each protein are presented as percentage of entire protein length and as absolute length in amino acids. Sgs = salivary glue protein, Dp = dumpy, Tnc = tenectin, (0.07 MB DOC) [file pone.0003041.s004.doc]

| Name *^1^ | Gene id | Genomic localization | Length (aa) | SS  (position; aa) | TM  (position; aa) | ST (%) | P (%) | RD(%) | RD(aa) |
| --- | --- | --- | --- | --- | --- | --- | --- | --- | --- |
| Mucins | | | | | | | | | |
| Muc14A | CG32580 | 14A6 | 16223 | 1-19 | - | 35.3 | 4.2 | 85.1 | 13806 |
| Muc12Ea | CG32602 | 12E7 | 3458 | 1-5 | - | 38.1 | 15.2 | 94.7 | 3275 |
| Muc68Ca | CG18331 | 68C15 | 3135 | 1-18 | - | 49.1 | 2.8 | 71.5 | 2242 |
| Muc30E | CG33300 | 30E1 | 1761 | 1-17 | - | 43.7 | 9.3 | 82.1 | 1446 |
| Muc25B (Sgs1) | CG3047 | 25B4 | 1225 | 1-22 | - | 59.5 | 13.9 | 71.0 | 870 |
| Muc91C | CG7709 | 91C1 | 950 | 1-32 | - | 32.3 | 19.5 | 55.9 | 531 |
| Muc55B | CG5765 | 55B2 | 485 | 1-17 | - | 28.7 | 14.6 | 51.5 | 250 |
| Muc4B | CG32774 | 4B4 | 483 | - | - | 50.7 | 9.5 | 41.4 | 200 |
| Muc68Cb (Sgs3) | CG11720 | 68C11 | 307 | 1-23 | - | 44.0 | 14.7 | 74.3 | 228 |
| Chitin binding Mucins | | | | | | | | | |
| Muc68E | CG33265 | 68E4 | 1799 | - | - | 32.9 | 7.6 | 77.4 | 1392 |
| Muc68D | CG6004 | 68D4 | 1514 | 1-19 | - | 42.5 | 6.0 | 54.6 | 827 |
| Muc11A | CG32656 | 11A2-11A3 | 1040 | 1-26 | - | 17.9 | 15.2 | 37.2 | 387 |
| Muc96D | CG31439 | 96D4 | 881 | 1-21 | - | 70.7 | 5.0 | 72.1 | 635 |
| Muc26B | CG13990 | 26B8 | 471 | 1-19 | - | 37.4 | 9.8 | 39.1 | 184 |
| Muc18B | CG7876 | 18B1 | 308 | 1-21 | - | 26.9 | 8.1 | 39.3 | 121 |
| Mucin-related proteins | | | | | | | | | |
| Mur24F (Dp) | CG33196 | 24F4-25A1 | 23015 | 1-21 | 22954 | 16.3 | 12.5 | 17.2 | 3959 |
| Mur96B (Tnc) | CG13648 | 96B15 | 2771 | - | - | 20.4 | 11.2 | 28.8 | 798 |
| Mur11Da | CG32644 | 11D5 | 582 | - | - | 43.6 | 8.8 | 29.2 | 170 |
| Mur82C | CG12586 | 82C2 | 559 | - | - | 30.9 | 13.2 | 22.0 | 123 |
| Mur29B | CG31901 | 29B1 | 555 | 1-24 | - | 54.4 | 1.3 | 65.4 | 363 |
| Chitin binding mucin-related proteins | | | | | | | | | |
| Mur89F | CG4090 | 89F1 | 2112 | - | - | 35.7 | 7.4 | 40.4 | 853 |
| Mur2B | CG14796 | 2B2 | 1795 | 1-21 | - | 33.3 | 10.1 | 1.9*^2^ | 34*^2^ |
| Mur18B | CG7874 | 18B1 | 481 | - | 28 | 26.0 | 7.5 | 22.7 | 109 |

*^1^Previous nomenclatures are provided in brackets.

*^2^= a long stretch of degenerate repeats (607aa) is present in *Mur2B.*
