# Supplementary material for: A Potential Role for Drosophila Mucins in Development and Physiology
Source: PLoS One. 2008 Aug 22;3(8):e3041. doi: 10.1371/journal.pone.0003041 (PMC2515642; doi:10.1371/journal.pone.0003041)
Supplement: Table S5 — List of oligos used for RT-PCR and for synthesis of the RNA in situ probes. For each gene, we used the same set of oligos for Reverse Transcription PCR and for amplification of the template DNA used to generate the in situ probes. When two different oligo pairs (A and B) are presented for a single gene, it means that the products yielded differential expression patterns in RNA in situ hybridization. (0.08 MB DOC) [file pone.0003041.s006.doc]

|  | Gene id |  | Forward primer | Reverse primer |
| --- | --- | --- | --- | --- |
| Mucins |  |  |  |  |
| Muc14A | CG32580 |  | CCTCGAGGACTCAAGAAGA | AGTTGATGTTGAGTCCGAAC |
| Muc12Ea | CG32602 |  | CTGTTCGTTATCTGCCTGCT | TCCAGTGTGGGCTTTGGTG |
| Muc68Ca | CG18331 |  | CTTGTTCCTCGTCGCGGTT | AGTTACATCTACGACTGGAG |
| Muc30E | CG33300 |  | ACTCTGCAAATGCAGCGCT | TGTTATTAGCTCAGATGTGGA |
| Muc25B (Sgs1) | CG3047 |  | TACGACTGGGATAGTATGCA | TCGATCAGAGCATGTGCACT |
| Muc91C | CG7709 |  | CAGCAGTGTAGTTCGCATTC | TCCATAGCTGGACGAGGGA |
| Muc55B | CG5765 |  | CTATTGGCCACCAGTGGCT | ATCCAAGCGAGCTGAATCCA |
| Muc4B | CG32774 |  | AACTGTGGGCCAGGAGTCT | GAATGGTAGAGGTCGACTGT |
| Muc68Cb (Sgs3) | CG11720 |  | TTGCAAGAGCTGCGGTCCT | TTCTTACACGTAAAATCAATTGA |
|  |  |  |  |  |
| Chitin binding mucins | | | | |
| Muc68E | CG33265 |  | ATGCTTCTGTGCAACTCGAA | GACAGTGGTAGTTTCCTCAG |
| Muc68D | CG6004 |  | CTGGGAAAGGACTTCTGTG | GTTGGGGATGAATCCGAAGA |
| Muc11A | CG32656 | A | GACTACTGGTGTTGCTAGTG | CTTCCGAACCTGTGGTAGT |
|  |  | B | GCTGTTCCGACTAAGCCATC | GAGGTGAATTGGGGTCAATG |
| Muc96D | CG31439 |  | ATGAGGAATGTATACGCTTTTG | CTGAGCACGGAGTTTGCGT |
| Muc26B | CG13990 |  | GAACAGCTGTCCATAGAATGA | ACATGGTACTGCGGCGATC |
| Muc18B | CG7876 | A | ATCAACGATCCAGGCCAAGT | ACTTTCTCGGTTGTGCCAG |
|  |  | B | AGTACCGATGAGCCCAACAC | AGGACACACCATCCATCCAT |
|  |  |  |  |  |
| Mucin-related proteins | | | | |
| Mur24F (Dp) | CG33196 |  | ATGGGTGTGCCACGGATAC | TGCCGTGATGACCAGTGCT |
| Mur96B (Tnc) | CG13648 | A | ACGAGTTGGACTTTATGGAC | TCTCTGGCGTAGTGATCTCA |
|  |  | B | GACAATTCCCGAAATCTCCA | CAGCATCCTGAGGAGACACA |
| Mur11D | CG32644 |  | TCGAATTGACAGAACCGAT | AGTTCTTCCGTAACATGGCT |
| Mur82C | CG12586 |  | ACTTCTTGGGGATGTTGAAC | ACCTCCATGGCAATAAGCTC |
| Mur29B | CG31901 |  | GTGCAGTGCGATTCCCATC | CTGTTGATGAATCTGTTGTAG |
|  |  |  |  |  |
| Chitin binding mucin-related proteins | | | | |
| Mur89F | CG4090 |  | AGACAGACGTGGCCTTCTAT | GATAAAACACGGTGCAGTCG |
| Mur2B | CG14796 |  | GAACCACGAGGATTCCAACT | TGGTCGCTGAGTAGGAGCA |
| Mur18B | CG7874 | A | CATTTACGGTTCGCAAGATG | AGCTGATGATGTCTGGTCTG |
|  |  | B | CTCCTGCAGAGCCAGAGAGT | CTCATCAATTCCGGCAAATC |
|  |  |  |  |  |
| Control |  |  |  |  |
| 18SrRNA |  |  | GAAGTATGGTTGCAAAGCTGA | TGTTGTAAGTACTCGCCACA |
